# Characteristics of Developmental and Epileptic Encephalopathy Associated with *PACS2* p.Glu209Lys Pathogenic Variant—Our Experience and Systematic Review of the Literature

**DOI:** 10.3390/biom14030270

**Published:** 2024-02-23

**Authors:** Adina Stoian, Zoltan Bajko, Rodica Bălașa, Sebastian Andone, Mircea Stoian, Ioana Ormenișan, Carmen Muntean, Claudia Bănescu

**Affiliations:** 1 Department of Pathophysiology, George Emil Palade University of Medicine, Pharmacy, Science and Technology of Targu Mures, 540139 Targu Mures, Romania; adina.stoian@umfst.ro; 2 Department of Neurology, George Emil Palade University of Medicine, Pharmacy, Science and Technology of Targu Mures, 540139 Targu Mures, Romania; rodica.balasa@umfst.ro (R.B.); sebastian.andone@umfst.ro (S.A.); 3 Department of Anesthesia and Intensive Care, George Emil Palade University of Medicine, Pharmacy, Science and Technology of Targu Mures, 540139 Targu Mures, Romania; mircea.stoian@umfst.ro; 4 1st Neurology Clinic, Mures County Emergency Hospital, 540142 Targu Mures, Romania; ioana.ormenisan@gmail.com; 5 Department of Pediatrics, George Emil Palade University of Medicine, Pharmacy, Science and Technology of Targu Mures, 540142 Targu Mures, Romania; carmen.duicu@umfst.ro; 6 Department of Genetics, George Emil Palade University of Medicine, Pharmacy, Science and Technology of Targu Mures, 540142 Targu Mures, Romania; claudia.banescu@umfst.ro

**Keywords:** PACS2, brain atrophy, neurological regression, epileptic encephalopathy, developmental and epileptic encephalopathy

## Abstract

Background: Developmental and epileptic encephalopathies (DEE) encompass a group of rare diseases with hereditary and genetic causes as well as acquired causes such as brain injuries or metabolic abnormalities. The phosphofurin acidic cluster sorting protein 2 (PACS2) is a multifunctional protein with nuclear gene expression. The first cases of the recurrent c.625G>A pathogenic variant of *PACS2* gene were reported in 2018 by Olson et al. Since then, several case reports and case series have been published. Methods: We performed a systematic review of the PUBMED and SCOPUS databases using Preferred Reporting Items for Systematic Review and Meta-Analyses (PRISMA) guidelines. Our search parameters included DEE66 with a pathogenic *PACS2* gene p.Glu209Lys mutation published cases to which we added our own clinical experience regarding this pathology. Results: A total of 11 articles and 29 patients were included in this review, to which we added our own experience for a total of 30 patients. There was not a significant difference between sexes regarding the incidence of this pathology (M/F: 16/14). The most common neurological and psychiatric symptoms presented by the patients were: early onset epileptic seizures, delayed global development (including motor and speech delays), behavioral disturbances, limited intellectual capacity, nystagmus, hypotonia, and a wide-based gait. Facial dysmorphism and other organs’ involvement were also frequently reported. Brain MRIs evidenced anomalies of the posterior cerebellar fossa, foliar distortion of the cerebellum, vermis hypoplasia, white matter reduction, and lateral ventricles enlargement. Genetic testing is more frequent in children. Only 4 cases have been reported in adults to date. Conclusions: It is important to maintain a high suspicion of new pathogenic gene variants in adult patients presenting with a characteristic clinical picture correlated with radiologic changes. The neurologist must gradually recognize the distinct evolving phenotype of DEE66 in adult patients, and genetic testing must become a scenario with which the neurologist attending adult patients should be familiar. Accurate diagnosis is required for adequate treatment, genetic counseling, and an improved long-term prognosis.

## 1. Introduction

Developmental and epileptic encephalopathies (DEEs) comprise a group of rare diseases with early childhood onsets characterized by clinical heterogeneity, associated malformations and developmental dsisorders of the central nervous system (CNS) [1], early onset epilepsy, recurrent seizures with interictal epileptiform activity, developmental slowing, intellectual disability, speech impairment, psychiatric problems, behavioral and autism spectrum disorders, and developmental regression [2,3]. Many patients present with cognitive deficits resulting from genetic defects and neurobiological changes and processes associated with the disease that alter normal childhood development [4,5,6]. Epileptic activity can also directly and negatively impact cognition, which is why appropriate intervention and in some cases aggressive treatment is needed to prevent further damage [4]. An increased frequency of epileptic seizures can contribute to cognitive regression, with alterations in intellectual acquisitions acquired during development. 

The etiology of DEEs is varied, involving both hereditary and genetic causes, as well as acquired causes such as metabolic abnormalities or brain injuries [7]. One characteristic of DEEs related to gene variants is the early onset of symptoms, beginning in the first days or weeks of life [4]. The term “DEE” is used when both epileptic activity and development disorders impact an individual’s behavior and cognitive status [5]. 

DEEs are defined especially by the pediatricians with the description of characteristic phenotype in children. As research and therapeutic modalities in medicine have advanced, the use of symptomatic and supportive treatments has led to an increase in life expectancy for these patients, some reaching adulthood and presenting anomalies of evolution over time that do not yet benefit from a complete description of the clinical picture. The epileptiform activity can vary depending on the age and stage of brain maturity, with phenotypic changes as age advances. The frequent seizures and epileptic electrical discharges may worsen the evolution of a DEE and are also responsible for behavioral, cognitive, and motor regression, with progressive neurological damage contributing to the aggravating psychomotor dysfunction [5,8].

Two PACS genes have been described in vertebrates: *PACS1* and *PACS2* [9]. The genes have different functions but similar structures [10]. The *PACS1* and *PACS2* genes are widely distributed in neuronal centers (*PACS1*); white matter glial cells, brain hemispheres, spinal cord (particularly the cervical region), peripheral blood lymphocytes [2,7], heart tissue, liver tissue, and skeletal muscle (*PACS2*) [3,11]. 

The *PACS1* gene, located on chromosome 11.q13.1–q13.2, consists of 24 exons. The *PACS2* gene, located on chromosome 14q32.33, consists of 26 exons. Both *PACS1* and *PACS2* mutations have been reported to be associated with neurological disorders: early onset epilepsy, intellectual disability, global developmental delay, and different malformations [12,13].

The *PACS2* gene encodes the protein Q86VP3, which consists of 889 amino acids. PACS2 is a multifunctional protein with nuclear gene expression, is well-expressed in the brain, is involved in communication between the endoplasmic reticulum membrane and mitochondria [1,9,14], and may be involved in ion channel trafficking [10]. *PACS2* c.625G>A is a missense mutation localized in exon 6. It leads to the replacement of glutamic acid (Glu or E) with lysine (Lys or K). The first cases of the recurrent c.625G>A (p.Glu209Lys) pathogenic variant of the *PACS2* gene with DEE 66 were reported in 2018 [7]. *PACS2* gene mutation is associated with intellectual development disorders, autism, early onset (predominantly neonatal), epilepsy, cerebellar damage, occasional facial dysmorphisms, hematological disorders, and, in rare cases, distal limb abnormalities [2,3,14]. 

The understanding of the molecular substrates driving the pathophysiology of neurological diseases that involve intellectual disability, neurodevelopmental disorders, and epileptic seizures has been greatly enhanced following the advent of whole exome sequencing and further advances in genome analysis [1]. There is a growing need for patients to receive an etiological diagnosis. The objective of this paper is to provide updated information to help with an accurate prognosis, appropriate treatment recommendations, and suitable genetic advice. 

## 2. Material and Methods

### 2.1. Literature Review 

#### 2.1.1. Information Sources and Search Strategy 

We reviewed the existing literature following the PRISMA guidelines (http://www.prima-statement.org, accessed on 5 December 2023). We conducted searches using the PUBMED and SCOPUS databases and several search terms: “*PACS2* gene”, “*PACS2* mutation”, and “developmental and epileptic encephalopathy 66”. Our aim was to clarify the challenges associated with DEE patients and the characteristics of epileptiform encephalopathy cases associated with the *PACS2* p.Glu209Lys pathogenic variant.

#### 2.1.2. Eligibility Criteria

We tried to identify previously published cases involving patients with DEE66 and a *PACS2* gene p.Glu209Lys mutation. Our analysis focused on neurological and psychiatric clinical manifestations, the presence of other symptoms or malformations in other organs, paraclinical examinations performed, and management strategies and treatments used. We also assessed the progression of the disorder and the clinical outcomes associated with therapeutic interventions.

Inclusion criteria: We applied several inclusion criteria to select studies. We only included English language in extenso articles or those with institutional access that presented case reports, case series, or reviews involving patients diagnosed with DEE66 who had undergone genetic testing to confirm the presence of the *PACS2* p.Glu209Lys mutation. We only included articles that contained relevant data. The articles and titles were evaluated by two independent experts (AS and MS), with their results compared to reach a consensus.

Exclusion criteria: The exclusion criteria included articles published in languages other than English, general reviews, articles containing insufficient data, those without full article access, and those involving a neurological disorder other than DEE66.

**Figure 1:**
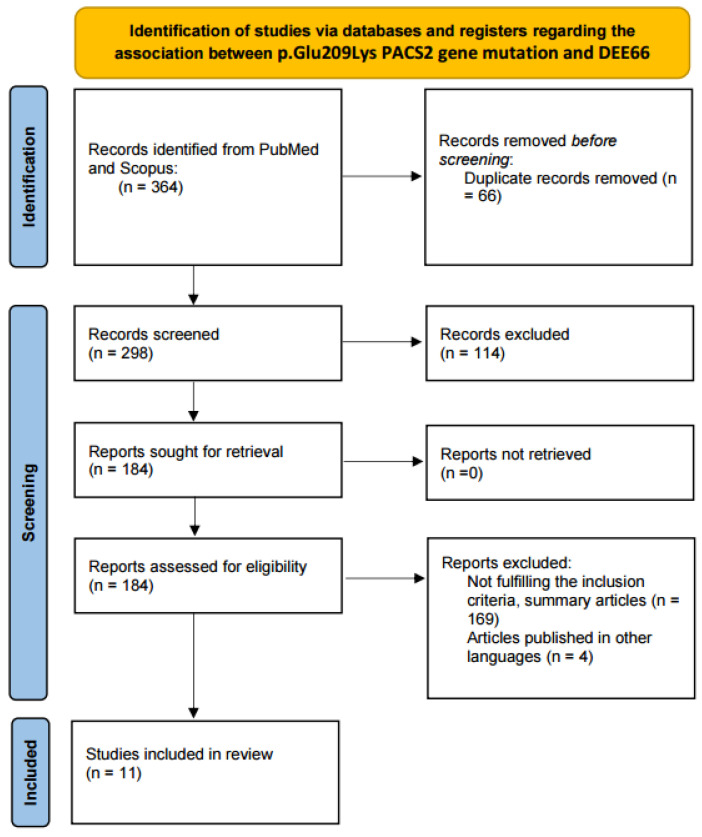
PRISMA flow diagram for the review.

All identified articles were analyzed and screened. The evaluated articles were published between 15 October 1999 and 6 December 2023. In total, 364 articles were identified using the search criteria. Following duplicate removal (n = 66), 298 records were screened and analyzed. Following this, 114 articles were excluded due to not containing relevant data. The remaining 184 articles were further assessed for eligibility. One hundred and sixty-nine either did not fulfill the inclusion criteria or did not allow full access, while four articles were published in languages other than English. Therefore, 11 publications were selected for inclusion. From these publications, 29 patients were identified and included in the review. We added our own case to this patient group. A flow chart of the search strategy is illustrated in Figure 1. 

The data extracted from the selected articles were sex, age at which epileptic seizures began, epileptic seizure characteristics, age at which a genetic diagnosis was established, neurological symptoms, psychiatric manifestation, presence of facial dysmorphisms or other anomalies in different organs or systems, electroencephalogram (EEG) characteristics, brain imaging investigations performed and the detected anatomical changes, type of treatment administered, and outcomes. The collected data are presented in Table 1. It includes the information on all patients with the *PACS2* p.Glu209Lys pathogenic variant and neurological disorders, except for those with incomplete data.

### 2.2. The Methodology Used to Examine Our Own Patient

A blood sample collected in an EDTA vacutainer was used for genomic DNA (gDNA) isolation. Next-generation sequencing (NGS) was performed using a hybridization-based protocol to enrich the target regions. Illumina technology was used for high-throughput sequencing. Based on the patient’s phenotype, a sequencing analysis was performed using an Invitae Leukodystrophy and Genetic Leukoencephalopathy Panel; however, no pathogenic variants were identified in the 697 genes analyzed (Invitae, San Francisco, CA, USA). Considering the cognitive and motor developmental delay demonstrated by the patient, the Invitae Neurodevelopmental Disorders (NDD) panel was additionally requested. The raw reads were aligned to the genome reference GRCh37 (Consortium Human Build 37), which is known as human genome assembly 19 (hg19). The patient was found to be heterozygous for the pathogenic variant c.625G>A (p.Glu209Lys or E209K) in the *PACS2* gene. Heterozygous *PACS2* gene mutations are reported to result in DEE66 (MIM #618067) [7]. The *PACS2* c.625G>A variant, also called rs1555408401, is not found in population databases, such as the Genome Aggregation Database (gnomAD). 

To predict the impact of glutamic acid (Glu or E) substitution with lysine (Lys or K) on the structure and function of the Q86VP3 protein, we used the PolyPhen-2 [24] algorithm, which predicted that this variant is “possibly damaging”, with a score of 0.757 (sensitivity: 0.85; specificity: 0.92). According to ClinVar, it is pathogenic/likely pathogenic [25], while based on VarSome, it is pathogenic [26].

We used the MutPred2 software to predict the pathogenicity of the *PACS2* E209K substitution by integrating molecular and genetic data [27]. The software predicted a gain of SUMOylation, ubiquitylation, and methylation at E209 and a gain of loop and phosphorylation at S213, with a MutPred2 score of 0.534 and *p* ≤ 0.05. We used the variant effect predictor (VEP) from Ensembl to investigate the effect of the identified variant on the *PACS2* gene, Q86VP3 protein sequence, transcripts, and regulatory regions identified in our patient [28]. A moderate impact was revealed. Other algorithms developed to predict the effect of missense changes on protein structure and function suggested that this gene mutation may result in a disrupted protein product (SIFT = deleterious; PolyPhen = possibly damaging [0.474]; CLIN SIG = pathogenic, likely pathogenic; CADD PHRED = 28.9; CADD RAW = 4.23). 

## 3. Results 

### 3.1. Literature Review

We included eleven articles in our review. Four were case series and reviews, and seven were case reports. Olson et al. reported 14 cases that included relevant data [7]. Chou et al. published a review of cases they identified from databases that included English, Chinese, and Japanese articles [22]. They included 29 patients in their review and described three patients of their own. We found relevant data when we assessed the descriptions of their patients. However, the data for some patients included in the review were insufficient. The total number of patients included in our review was 30, including our own patient.

Most of the cases included (53.33%) were male patients, but there was not a significant difference between sexes (M/F: 16/14), and the age of seizure onset varied between 1 day and 10 months old. However, in most cases (76.66% (23/30)), the seizures began in the first 2 weeks of life. Our own patient was male, and his seizures were first noticed at 4.5 months old. A varied range of seizure manifestations were observed among the cases, including focal motor seizures, grasping, tonic seizures, autonomic manifestations, tonic-clonic seizures, abnormal eye movements, and myoclonic seizures. The age of genetic diagnosis varied between 4 months and 37 years. Our patient’s genetic diagnosis was made at 31 years old.

The most common neurological and psychiatric symptoms found in the reviewed cases were speech delay (19 cases), hypotonia (17 cases), delayed global motor skills and developmental delay (24 cases), behavioral disturbances (15 cases), nystagmus (4 cases), pyramidal syndrome (4 cases), and wide-based gait (4 cases). Our patient presented with one and a half syndrome, reduced visual acuity, facial hemi spasm, pyramidal syndrome, truncal ataxia, wide-based gait, nystagmus, and global developmental delay. The common behavior features included social isolation with behavioral disturbance, limited verbal communication, and global (including intellectual) developmental delay. Our patient presented with motor delay during childhood, limited verbal speech, reduced social communication, reduced intelligence quotient (IQ), and limited intellectual capacity. 

The most commonly reported facial anomalies and dysmorphisms included hypertelorism, broad nasal roots, thin upper lips, highly arched eyebrows, long eyelashes, wide-spaced teeth, and down-turned lip corners. Facial dysmorphisms were not overly evident in our patient, except for a thin upper lip and slightly spaced teeth. Other symptoms and organ involvement included limb distal malformations and changes (12 cases), ocular abnormalities (strabismus, nystagmus, hypermetropia, astigmatism, myopia, coloboma) (11 cases), hematological disturbances (5 cases), cryptorchidism (5 cases), cardiac (atrial and ventricular) septal defects (4 cases), hydronephrosis (2 cases), reduced growth velocity (1 case), knee arthritis (1 case), horseshoe kidneys (1 case), repetitive urinary infections (1 case), bilateral vesicoureteral reflux (1 case), ectopic testis (1 case) and gastroesophageal reflux (1 case). Our patient presented with reduced visual acuity and cryptorchidism. 

The EEG epileptiform abnormalities varied among the patients, with focal or multifocal onsets observed. Some cases involved generalization, burst suppression or hypsarrhythmia, local or multifocal irritative interictal discharges (spikes), diffuse cortical interictal dysfunction, or EEG normalization over time. 

Brain MRI scans were the most commonly performed radiological investigation. Abnormal neuroimaging findings were observed including dysgenesis of the cerebellar folia (15 cases), mega cisterna magna (13 cases), inferior vermis hypoplasia (8 cases), white matter reduction and prominent extracerebral CSF spaces (6 cases), lateral ventricles asymmetry and ventricle enlargement (4 cases), hypothalamic fusion anomalies (3 cases), hyperintensities located in the white matter, midbrain and surrounding lateral ventricles (3 cases), mesial temporal sclerosis (1 case), and cavum septum pellucidi (1 case). The MRI scan of our patient revealed abnormal cerebellar foliation, vermis hypoplasia, hyperintensities in the white matter, enlarged lateral ventricles, cavum septum pellucidi, enlarged extracerebral CSF spaces, and severe cerebral and cerebellar atrophy. 

Different medications and therapeutic schemes with different drug combinations were used to control the seizures, and some of them were reported to have efficacy, such as valproic acid, levetiracetam, oxcarbazepine/carbamazepine, and vigabatrin. Our patient first received valproic acid, followed by levetiracetam and then brivaracetam. This treatment demonstrated good seizure control, with the patient being seizure-free. However, this treatment did result in the occurrence of a left facial hemi spasm. Clonazepam was administered successfully.

The reported data revealed more difficulty associated with controlling epileptic seizures in childhood, with multiple drug combinations and changes in treatment schemes required. However, with advancing age, the seizures became easier to control with medication or even disappeared. The patients presented with intellectual disability, behavioral disturbances, reduced IQs, and poor social communication. Our case had all these characteristics. 

### 3.2. Our Clinical Experience 

Our patient is a 31-year-old male who is the only child of intellectual, non-related, and healthy parents. No significant familial diseases are known, and the patient was born full-term following an uneventful pregnancy. No fetal suffering was noted during birth or pregnancy, and the patient demonstrated growth parameters in the normal range prenatally and postnatally. He developed tonic seizures at 4.5 months old. Following the first epileptic seizure, viral encephalitis was suspected. A lumbar puncture (LP) was performed for cerebrospinal fluid (CSF) analysis, and antiepileptic treatment using valproic acid was introduced. This resulted in a reduction in seizure frequency. No pathological changes in the CSF and no pathogenic etiological agent were identified. The patient started walking unassisted at approximately 2 years old and demonstrated a slower acquisition of language. The patient had cryptorchidism, which was surgically corrected at the age of 5, and was diagnosed with a neurocognitive developmental disorder involving psychomotor retardation. 

The patient was educated and received poor results in the exact sciences. He received help from his parents, particularly his mother, and demonstrated the ability to acquire some knowledge related to history and geography. He attended a school that provided tailored support. He attempted to complete higher education lectures in history and was constantly supported by his mother, who studied with him. However, he never practiced in the field. 

During the disease’s evolution, the neurologists who monitored the patient’s epileptic seizures replaced valproic acid with levetiracetam, then with brivaracetam. No tonic-clonic seizures were observed with the last therapeutic scheme; however, a left facial hemi spasm was noted. From a behavioral point of view, the patient tends to isolate himself, demonstrates behavioral stereotypes and reduced spontaneous and expressive language, and is not able to describe his symptoms or reasons for hospitalization. 

He was admitted to our clinic due to progressive involuntary weight loss (approximately 10 kg over 3 years), decreased muscle strength, left facial hemi spasms, sudden-onset divergent strabismus, dizziness, gait instability, marked accentuation of social isolation, and cognitive decline, particularly in the context of COVID-19 pandemic. The clinical picture apparently did not fit with any known neurological disease. 

The neurological examination at admission revealed one and a half syndrome with left abduction nystagmus during lateral eye movements, myopia with reduced visual acuity, left facial hemi spasms, truncal ataxia, flaccid tetra paresis (grade 4/5 on the Medical Research Council [MRC] scale), inconstant bilateral Babinski sign without evident sensory deficits, bradypsychia, bradylalia, echolalia, and a mini-mental state examination (MMSE) result of 17 points out of 30.

The usual blood tests were performed, including autoimmune disorder screening and infectious disease screening. Ceruloplasmin, serum copper, calcium, and angiotensin-converting enzyme levels were within normal limits. An insufficient level of vitamin D (22.4 ng/mL; normal range: 30–55 ng/mL) was noted. An LP for CSF examination was conducted, with no pathological changes or neurological status improvements observed. Oligoclonal bands were absent in the CSF.

**Figure 2:**
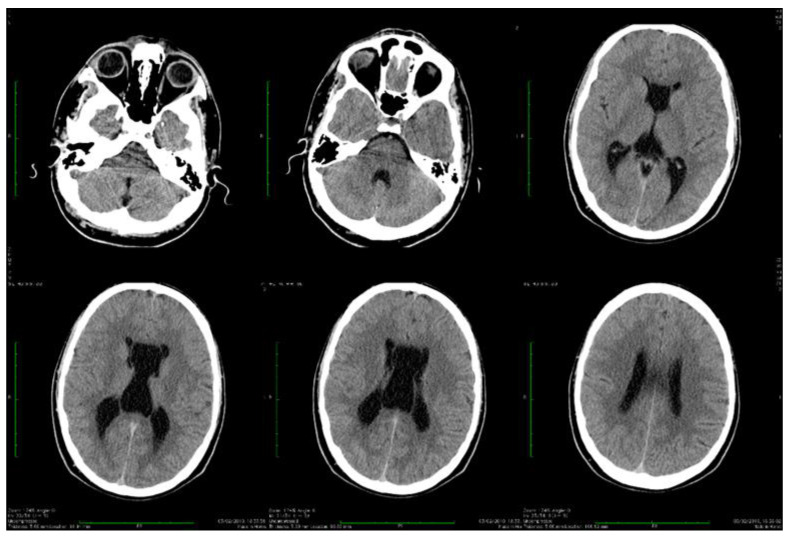
Axial computed tomography scans showing wide cavum septum pellucidum and enlargement of the lateral ventricles (2009).

**Figure 3:**
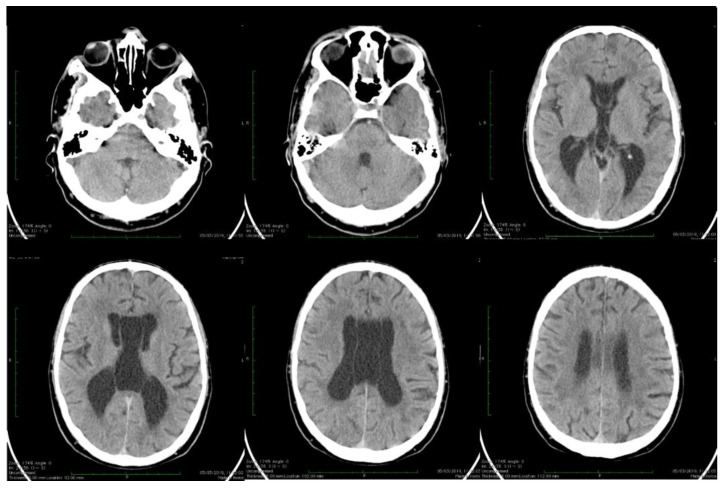
Axial computed tomography scans showing wide cavum septum pellucidum and enlargement of the lateral ventricles (2019). The evolution of cerebral atrophy in ten years is evident on serial brain CT scans.

**Figure 4:**
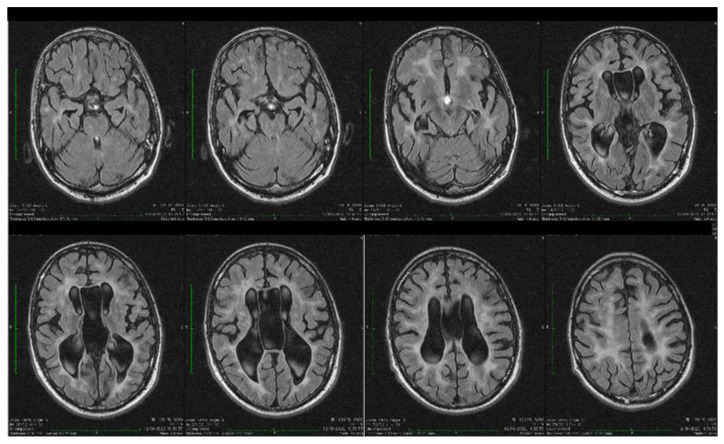
Axial fluid attenuated inversion recovery (FLAIR) MRI sequences showing wide cavum septum pellucidum, enlargement of the lateral ventricles, diffuse cerebral and cerebellar atrophy, and periventricular white matter hyperintensities. The brain atrophy is even more evident compared with the previous brain imagistic investigations.

**Figure 5:**
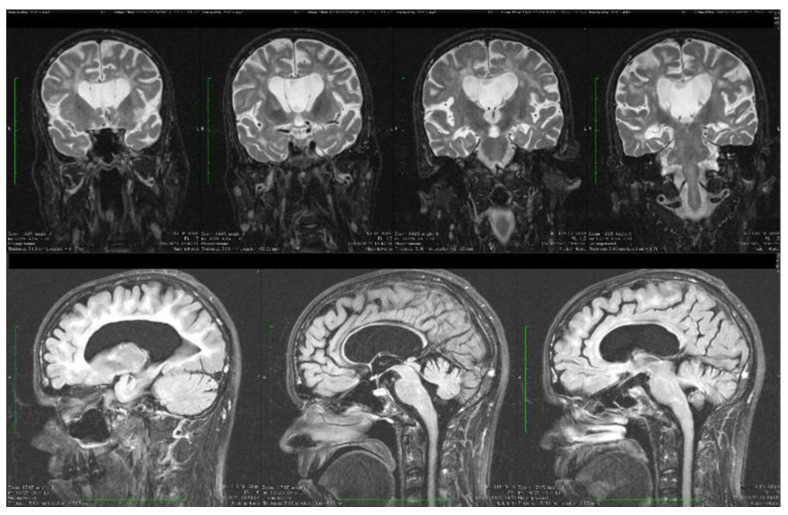
Coronal and sagittal T2 and FLAIR MRI sequences showing wide cavum septum pellucidum, enlargement of the lateral ventricles, diffuse cerebral and cerebellar atrophy, and periventricular white matter hyperintensities.

Dynamic evaluations using radiological examination were requested by various neurologists when the patient was 18 and 28 years old. The findings revealed a progressive white matter disorder with cerebral atrophy and progressive enlargement of the CSF spaces. The CT scan performed 13 years and 3 years prior to his admission to our clinic revealed generalized progressive sufferance of white matter with diffuse chronic hypodensities up to 1 cm in diameter (that were more evident at the last brain CT scan examination) at the level of the right internal capsule and lenticular nucleus, cavum septum pellucidi, an enlarged ventricular system, and progressive cerebral atrophy (Figure 2 and Figure 3). The MRI scan performed just before admission revealed severe cerebral atrophy, demyelinating lesions in the pons, middle cerebellar peduncles, supratentorial white matter with chronic lesions in the right and left temporal lobes and the right frontal and parietal lobes, marked atrophy of the right hippocampus in the context of general cerebral atrophy, cavum septum pellucidi, and severe enlargement of the CSF spaces. However, no radiological signs of active hydrocephalus were noted (Figure 4 and Figure 5). 

An EEG recording was performed while the patient was awake; performing this was difficult due to poor patient cooperation. The EEG revealed poor, unmodulated alpha activity that was more evident in the right posterior derivations, not modulated in the spindles, and with no changes when the eyes were opened. Intermittent theta activity was also observed. There were sharp wave discharges, with sharp peaks on the left central, parietal, and temporal derivations.

Bilateral nerve conduction studies were performed using a four-channel electromyography (EMG) machine (Nihon Kohden, Neuropack MEB-9400, Tokyo, Japan) and surface electrodes to assess the peripheral nervous system, including the median, ulnar, peroneal, tibial, and sural nerves. No peripheral nervous system issues were observed. A cardiac and abdominal ultrasonographic examination did not reveal any abnormalities. An ophthalmological examination was carried out and highlighted low visual acuity, for which the patient already had an indication for optical correction. 

Due to the evolutionary nature of the disease, evidenced by epileptic seizures starting in early childhood, developmental deficits, and psychomotor retardation, as well as the radiological appearance, epileptiform leukoencephalopathy was suspected. We contacted the genetic department for counseling due to the high suspicion of a genetic disorder.

The genetic testing was performed only for the patient. We are most likely dealing with a de novo mutation. 

## 4. Discussion

### 4.1. DEE66 General Considerations

DEE66 is a neurological disease that manifests during the first days of life. It is associated with global developmental delays and CNS malformations. The early neonatal onset of epileptic focal seizures with secondary generalization can be difficult to control with drug treatments. Individuals with DEE66 often present with cerebellar dysgenesis, behavioral and neurophysiological developmental abnormalities, facial dysmorphisms, psychomotor retardation, behavioral disorders, hypotonia, facial dysmorphisms, such as ophthalmological defects, and, in some cases, extra neurological manifestations, such as hematological abnormalities (anemia, neutropenia), cryptorchidism, and distal limb abnormalities [7,29]. Congenital heart disease, ocular abnormalities, and distal limb anomalies have also been described in these patients [1,7]. 

Genetic testing for *PACS2* was first reported in 2018 by Olson et al. [7], who published the first study describing 14 unrelated individuals affected by a molecular spectrum of *PACS2* mutations that resulted in intellectual disabilities with paroxysmal epileptic manifestations. In these cases, the manifestation onset began in the first days or weeks of life. The authors demonstrated the existence of the recurrent de novo heterozygous missense variant G.625>A (p.Glu209Lys) in the *PACS2* gene [7]. 

Chou et al. [22] also performed a literature review of articles published in English, Chinese, and Japanese. In March 2023, they published an article reviewing 31 cases of the heterogenous missense variant *PACS2* p.Glu209Lys and one case of the heterogenous missense variant *PACS2* pGlu211Lys. One case included in their review and originally published by Valenzuela et al. [17] had inherited the p.Glu209Lys variant from the mother, who also presented the characteristic phenotype of this mutation. Most patients in their review were females (63%), and the onset of most epileptic seizures occurred in the perinatal period, with only one case reporting an onset at 2 months old [22]. We included in our review and in Table 1 only patients that presented the p.Glu209Lys *PACS2* gene mutation and did not include *PACS2* p.Glu211Lys missense mutations.

Motor developmental delay and walking difficulties were frequently reported by different authors [1,7,15,22] without an involvement of the peripheral nervous system [30]. Some patients presented with a wide-based gait [7,22]. This was also noted in our patient. Ocular abnormalities and the presence of nystagmus were also described [7,22]. This neurological sign was also present in our patient. Autism spectrum disorders and intellectual deficits with learning disabilities are characteristic of *PACS2*-related DEE and were reported by many authors, including us. The epileptic seizures described in the patients had varied morphologies, were focal, and were sometimes accompanied by autonomic nervous system manifestations or generalized seizures [7,22]. 

Brain imaging highlighted frequent anomalies of the posterior cerebral fossa with cerebellar dysplasia, cerebellar gyri irregularities, foliar distortion of the cerebellum, mega cisterna magna, cerebral atrophy, and vermis hypoplasia [7,22]. Some of these were present in our case. The vermis receives sensitive afferents from the lower limbs, trunk, upper limbs, cephalic extremity, and the vestibular and auditory apparatus and in turn sends efferent fibers to the cortex and brainstem [31]. Vermis atrophy and ataxic gait have been described in chronic alcohol users [32]. In our patient, rapidly progressing severe cerebral atrophy was evident at a young age. The brain CT scan performed in 2009 revealed generalized white matter disorder with diffuse hypodensities, ventriculomegaly, and the presence of cavum septi pellucidi. The brain MRI scan performed in 2022 highlighted the rapid progression of cerebral, cerebellar, and vermis atrophy, with cerebral hyperintensities similar to demyelinating lesions present in the pons, middle cerebral peduncles, bilateral hemispheric supratentorial white matter (without fulfilling the demyelinating or autoimmune CNS disease criteria), and marked right hippocampus atrophy [33,34,35,36]. 

Most cases initially responded to a single antiepileptic drug, only to subsequently relapse and require a drug change, with multiple therapeutic combinations sometimes used [7,22]. The treatment regimen changed over time in our patient. Most cases reported by Chou et al. [22] were treated with valproate or levetiracetam (~50%), phenobarbital (~33%), and carbamazepine (~25%), and seizure frequency appeared to decrease with age [7,22]. Perulli et al. reported an amelioration and disappearance of status epilepticus after intravenous pyridoxine supplementation [21]. The cases reported by Chou et al. [22] initially responded to intravenous pyridoxal phosphate. However, seizure recurrence occurred when the treatment was shifted to oral vitamin B6 [22]. Sánchez-Soler et al. questioned the usefulness of valproic acid due to the intensification of epileptic seizures observed following its administration [20].

Our case demonstrated mild facial dysmorphism, early onset epilepsy, and cerebellar dysgenesis, with developmental impairment and cognitive disorders that became more obvious with age.

The clinical characteristics and the evolution of our case present a phenotype that does not fit any previously known disease, which was the primary reason genetic counseling was sought. Our case fits into the characteristic phenotypic traits of the p.Glu209Lys *PACS2* gene mutation, which is characterized by early onset epileptic seizures in the neonatal period or in the first months of life, intellectual deficits, cerebellar dysgenesis, a tendency to socially isolate, minimal linguistic baggage, and autism spectrum disorders. Several clinical and imaging characteristics described in patients with DEE66 were also observed in our patient, including epileptic seizures with onset in the first months of life, psychomotor retardation, behavioral disorders, gait ataxia, ventriculomegaly, ophthalmologic alterations, and genital abnormalities (cryptorchidism). Other anomalies frequently encountered that were not detected in our patient include hematological alterations, distal limb abnormalities, and cardiac structure alterations. Among the included patients in this review, most of the epileptic seizures occurred in the neonatal period. It started between 1-day post-birth, with the latest onset described as 10 months old [7,22]. Unlike the majority of these cases, our patient also had a later onset, with seizures beginning approximately 4.5 months after birth.

Recent studies support the role of some DEE genes in neurodegeneration [37,38], and the evolution in adult patients with the *PACS2* p.Glu209Lys pathogenic variant suggests that this gene is also involved in neuronal loss and early neurodegeneration. The first reported case with cortical anomalies (polymicrogyria and pachygyria) belongs to Checri R et al. [23]. White matter reduction in the parietal region was described by Dentici et al. [1]. Brain atrophy at young ages and a decrease in posterior periventricular white matter volume can be observed in the images published by Mizuno T et al. [18]. Valenzuela et al. also describe a case with severe ventricular enlargements [17]. We want to point out that abnormal cortical aspects also occur in our patient, with severe cerebral atrophy suggesting that cortical development and neuronal survival during a patient’s lifetime, together with the dysregulation of the ion channels, might be influenced by this *PACS2* p.Glu209Lys pathogenic variant.

Hyperintensities in the frontal region were described by Dentici et al., and hyperintensities in the periventricular white matter were described by Perulli et al., similar to our findings [1,21].

As far as we know, ours is the first case of DEE66 with a *PACS2* p.209Lys mutation diagnosed and published in Romania and the first case diagnosed and reported at such an advanced age. Limited data are available regarding the life expectancy and clinical evolution of these patients. However, based on our patient’s evolution, we could assume that after the initial and delayed psychomotor development, intellectual degradation and loss of cognitive functions increase with age, and the patients are likely to have a developmental regression without being able of self-care. These data, combined with the radiological imaging investigations of our patient, suggest that nerve cell loss and structural alterations are more pronounced in these patients compared to the general population.

Compared to the phenotypes of previously reported cases, our patient presented with milder symptoms, delayed onset of epileptic seizures, and initially slightly better intellectual acquisitions. However, as he aged, a dramatic reduction in his intellectual capacity was noted. Our case highlights the importance of genetic testing to determine the correct diagnosis when epileptogenic hereditary leukoencephalopathy is suspected due to a clinical picture dominated by intellectual disability, epileptic seizures, and other neurological symptoms. Furthermore, due to the patient being 31 years old, the reporting of this case offers important data regarding the evolution of such patients.

**Figure 6:**
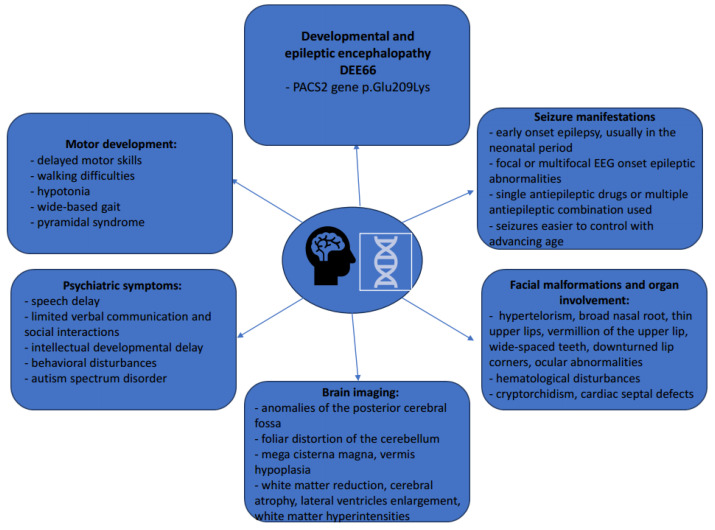
Clinical, phenotypical, and radiological characteristics of DEE66 patients.

The most important characteristics of patients with DEE66 are summarized in Figure 6.

Based on the clinical and radiological picture described in this review, children with phenotypic characteristics and specific radiological changes have begun to be tested more and more. Since 2018, several case reports and case series have been published. The information about these children’s clinical evolution and treatment was obtained retrospectively by accumulating case-by-case evidence once with their publication in medical journals. This pathology is not frequently encountered and is much underdiagnosed due to the lack of genetic testing. This attitude is not valid in adults, where a large number of cases might carry the *PACS2* p.Glu209Lys pathogenic variant but are not tested and properly diagnosed.

For this reason, information on the long-term evolution of these cases until adulthood is almost absent in the medical literature. In this review, we identified only three cases reported in adults, one with a very brief description, to which the case from own clinical experience is added [16,17,19]. Sakaguchi Y et al. described a 23-year-old man with initial motor development delay and progressive atrophy of the cerebellum [16]. Valenzuela et al. described a 25-year-old male with lateral occipital horns ventricular enlargement, which was interpreted as secondary to an obstruction produced by a vascular malformation [17]. The third case tested in adulthood and mentioned by Cesaroni et al., unfortunately, does not benefit from a detailed neurological examination or a radiological investigation performed at adult age and does not provide us with much information except for the fact that a woman carrying *PACS2* p.Glu209Lys pathogenic variant can give birth to children and pass the mutation on [19]. Our case appears to be the oldest reported with a complete description of the neurological exam and serial radiological investigations. The last radiological investigation carried out on our patient (at the age of 31) highlights an aspect of marked ventriculomegaly without signs of periventricular activity (which could be suggestive of active hydrocephalus), an aspect that is similar to the previously reported case. The question that arises is whether the ventricular enlargement described by Valenzuela et al. is secondary to deficient venous drainage at the level of cerebral circulation or whether hydrocephalus could be a characteristic of the natural evolution of the disease in adult patients with progressive cerebral atrophy and marked accelerated neuronal loss throughout life, leading to the occurrence of a dilated ventricular system as a consequence. The radiologic cerebral appearance described in our patient reinforces this assumption. 

**Figure 7:**
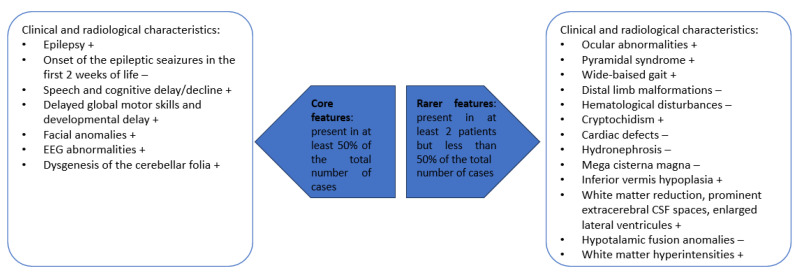
Features that are present in patients with p.Glu209Lys *PACS2* gene mutation; + present in our own patient; − absent in our own patient.

To summarize, the most important core and rare features characteristic for patients with p.Glu209Lys *PACS2* gene mutation are presented in Figure 7.

### 4.2. DEE66 Molecular and Pathophysiologic Considerations

PACS1 encodes a trans-Golgi membrane traffic regulator that is strongly expressed during the embryonic development of the brain [1,7] and is involved in directing phosphorylated cargo molecules [7,39]. PACS1 mutations have been described in Scuurs-Hoeijmakers syndrome, a known DEE [40]. In 2012, two cases were described secondary to a PACS1 mutation, which involved intellectual disability, epilepsy, facial dysmorphism, and generalized hypotonia [40]. 

*PACS2* encodes a multifunctional sorting protein that plays a vital role in pathway traffic regulation [7,39], ion channel trafficking, nuclear gene expression [41], autophagy, and apoptosis [10,12,42]. The missense variant G.625>A (p.Glu209Lys) in the *PACS2* gene is associated with phenotypic traits that partially overlap with the PACS1-related phenotype [7]. Different mutations in the autoregulatory domain of this gene can reduce its capacity to modulate the interaction of the *PACS2* protein with other proteins that participate in neuronal development and function [1,7]. CNS malformations are a common feature seen in patients with *PACS2* gene mutations [1,7].

Mitochondria-associated membranes (MAMs) consist of a 10-to-30-nm-wide cellular compartment formed by endoplasmic reticulum (ER) and mitochondria and play essential roles in physiological cellular processes and mitochondrial functions (e.g., Ca^2+^ homeostasis, lipid and phospholipid trafficking, inflammation and cholesterol metabolism) by establishing connections between mitochondria and the ER. MAMs enable a communication bridge between the plasma membrane, mitochondria, ER, and lysosomes [43,44,45,46], and are involved in apoptosis and autophagy processes [43,47,48,49,50]. Different phosphorylation events regulate the MAMs’ structure and function. The casein kinase 2 alpha 1 (CK2A1) is a catalytic subunit of casein kinase 2 and participates in the CK2A1-PACS-PKD2 complex. PKD2 is a non-selective cation channel with Ca^2+^ permeability on the ER membrane, mediating the mitochondrial Ca^2+^ entry [49]. The *PACS2* missense mutations (c.625G>A and c.631G>A) located in the CK2A1-phosphorylatable acidic clusters interfere with PKD2-dependent Ca^2+^ transfer between ER and mitochondria and alter the mitochondrial Ca^2+^ entry and the functional integrity of MAMs [49,51,52,53]. The PACS2 protein plays an important role in regulating the functionality of the outer mitochondrial membrane [43,46]. PACS2 is a traffic modulator that controls communication between the mitochondria and ER, ensuring the proper functioning of the ER, mitochondria, and lysosomes [1,22]. It interferes with the subcellular distribution of different proteins by binding with different cargo proteins, impacting their transfers to organelles and regulating different cellular activities. MAMs maintain intracellular homeostasis and the Ca^2+^ trafficking and crosstalk equilibrium between the mitochondria and ER [11,54]. Its disruption leads to cellular damage and apoptosis [43,55,56]. 

Reduced mitochondrial Ca^2+^ uptake is followed by decreased mitochondrial ATP production and a shortage of mitochondrial energy, facilitating catabolic processes and autophagy [49]. An overload of Ca^2+^ ions in the cytosol of the presynaptic nerve terminals leads to an increased presynaptic neurotransmitter release in glutamatergic neurons, which can explain the epileptic manifestations [49,57].

PACS2 is also considered to mediate death-ligand induced apoptosis produced by tumor necrosis factor (TNF)-related apoptosis-inducing ligand (TRAIL), which in vivo is a metastasis inhibitor that kills cancer or infected cells [12,58]. This action requires the permeabilization of different organelles, such as mitochondria and lysosomes [12,59], in which PACS2 plays an essential role. 

PACS2 has three important regions:

(1) The N-terminal furin binding region (FBR) can recognize and bind with various cargo proteins. It plays a role in intracellular protein trafficking by recognizing acidic amino acid residues on cargo proteins [60]. The *PACS2* p.Glu209Lys mutation leads to reduction/loss-of-function-like of PACS2 and alters the capacity of this region to form reversible bindings, disturbing cellular homeostasis [13].
(2) The middle region (MR) contains phosphorylation sites and plays a major regulatory role. It promotes interactions between proteins and regulates PACS2 functions during cellular growth, repair, and death [46]. This region contains a binding site that becomes active after phosphorylation for 14-3-3, forming together a complex that reduces tumor necrosis factor (TNF)-related apoptosis-inducing ligand (TRAIL) levels, inhibits apoptosis, and promotes lipid biogenesis [19,61].
(3) The C-terminal region (CTR): the specific function of CTR remains unclear [13,46].

Some cargo proteins that bind with PACS2 might induce the activation and recruitment of lysosomes, contributing to apoptosis [62]. Therefore, through its involvement in membrane trafficking, PACS2 plays an important role in maintaining tissue homeostasis through its proapoptotic and antiapoptotic effects [43]. The mutation of the *PACS2* gene results in alterations in the capacity of the autoregulatory domain to modulate this interaction, as well as interactions between PACS2 and other proteins that participate in normal neuronal function and brain development [1,7]. Other important functions include its role in the endosomal trafficking of different receptors and enzymes, and it has the capacity to induce apoptosis in response to death ligands by initiating mitochondrial membrane permeabilization and lysosomal activation [19,62]. In rodent experiments, the noradrenergic cell group A7 demonstrated that PACS-2 depletion in these cells prevents apoptosis by preventing cytochrome C release in the cytoplasm. Cytochrome C leads to the release of Ca^2+^ from the ER and activates executioner caspases [9]. Additionally, the selective apoptosis of tumor cells and virus-infected cells induced by TNF-α requires PACS-2 [8,59]. Some molecules, such as the cellular inhibitor of apoptosis, which is found in hepatobiliary cancer cells, promote PACS2 degradation, resulting in apoptosis inhibition [63]. However, in cases of DNA damage, PACS2 phosphorylation plays a cytoprotective role, highlighting its antiapoptotic role [43,59,64]. 

The deleterious and protective abilities of PACS2 depend on the type of stimulus involved and vary among different cells [43]. The *PACS2* p.Glu209Lys mutation [14] affects the capacity of the PACS2 FBR to bind cargo proteins [7], resulting in altered control over the interaction between cargo binding regions and proteins, causing alterations in cellular functions [7,11]. The c.625G>A missense mutation (referred to as E209K) occurs in the MR of *PACS2* and modifies the biochemical characteristics of this region. The p.Glu209Lys mutation alters the regulatory domain, reflected by difficulties in PACS2 binding cargo proteins, which negatively impacts neuronal migration, neurogenesis, interneural communication, cerebellum development, and dendritic arborization [65,66]. It is thought that mutations in this gene could cause ion channel dysfunctions, resulting in a phenotype that is similar to channelopathies that cause epileptic seizures. This could explain the favorable effects observed using drugs that act on voltage-gated sodium channels, such as carbamazepine [20,67,68].

Individuals with the *PACS2* c.625G>A missense mutation present with epileptic seizures early after birth and associated craniofacial and CNS malformations [7]. The neurological and psychiatric symptoms that occur in cases of mutation E290K were labeled by the National Institute of Health (NIH) MedGen database as DEE66 and result from imbalances in the cellular homeostasis of the nervous system due to dysregulated turnover of PACS-2 E290K, resulting in an increased protein half-life [69]. 

The 14-3-3 protein family, particularly 14-3-3Ɛ, can bind with signaling proteins and change their enzymatic properties, modulating neurodevelopment. Zang et al. demonstrated that compared with the PACS2 wild type, the presence of E209K mutations leads to an increased association with 14-3-3Ɛ, and this interaction correlates with increased susceptibility to apoptosis through disrupted signaling [9]. The presence of this complex might mediate proapoptotic signals during the period of neuronal development via cerebellar dysgenesis and morphological changes in the cerebellum [70,71,72]. The long-term evolution of our case, characterized by marked cerebellar and supratentorial progressive atrophy, suggests that proapoptotic signals also act later in the disease evolution, during the intrauterine life of the fetus, after birth, and during childhood, adolescence, and adulthood.

The oldest patients with the *PACS2* p.Glu209Lys pathogenic variant were described in the third and fourth decades of life. Zhang et al. asked themselves if the PACS2 protein dose could be related to developmental periods and the disorders that result from this fact [13,73]. We extend this question to the postpartum period and wonder if the subsequent survival of neurons could be related to the PACS2 protein. The neurologist who attends adult patients must gradually recognize the distinct evolving phenotype of DEEs. Sometimes, this may be challenging, but the correct identification of the diagnosis can increase the chances of adequate treatment. Gradually, genetic testing in adults must become a scenario with which adult neurologists should become familiar. A deeper understanding of the pathophysiological changes underlying gene defects will be helpful to pave the way toward future personalized treatments. Further research (which should include a larger cohort of patients of different ages) is needed for a better understanding of the disease and to widen the pool of knowledge. The mechanism linking rare gene mutations to brain development and accelerated neuronal death should be explored in more detail. 

## 5. Study Limitations

Only articles published in English to which we had free or institutional full access were included. Not all studies were well documented. Therefore, the number of patients evaluated in this review is small. There are only four adult patients (including our own) with the *PACS2* p.Glu209Lys pathogenic variant described. No extensive data is available regarding adults with this mutation. Future studies and free access to genetic testing could increase the number of reported cases.

## 6. Conclusions

The presence of the described clinical picture, combined with the characteristic phenotype and corresponding radiological changes should direct neurologists to engage a genetic specialist to obtain a correct diagnosis, even in adults. The reporting of some adult cases with this *PACS2* gene mutation gives us a perspective on the long-term evolution of these patients. Studying the natural history of the disease as the diagnosed children evolve into adults helps us to understand its physio-pathological mechanisms and can help develop personalized therapies. This pathology could be more common but underdiagnosed due to limited access, particularly due to the increased costs associated with genetic testing in countries where health insurance does not cover them, such as Romania. 

## Figures and Tables

**Table 1:** Summary of the literature data about all patients with *PACS2* p.Glu209Lys pathogenic variant and neurological disorders. NA means Non Available.

Authors	Study Design	Sex/Mean Age at Seizures Onset	Seizures Characteristics	Age at Genetic Diagnosis	Neurological Symptoms	Eye Features and Facial Abnormalities	Psychiatric/Behavioral Features	Other Symptoms and Organ Involvement	EEG	Imagistic Investigations and CNS Alterations	Treatment	Outcome
Olson et al. [7], 2018	Case series (Case 1)	Female/6 days	Focal seizures	16 years	Moderate myopia, delayed motor skills, hypotonia, increased tendon reflexes	Synophris, hypertelorism, down-slanting palpebral fissures, thin upper lip, down-turned corners of the mouth, prominent incisors and widely-spaced teeth, everted vermillion of lower lip	Speech delay	Neutropenia, metatarsus varus	Not described	Brain (magnetic resonance imaging) MRI: mega cisterna magna, foliar distortion of the left cerebellar hemisphere	Carbamazepine	Not described
Olson et al. [7], 2018	Case series (Case 2)	Female/4 days	Generalized tonic-clonic seizures	4 years	Delayed motor skills, nystagmus, wide-based gait,	Synophris, hypertelorism, down-slanting palpebral fissures, broad nasal root, thin upper lip, down-turned corners of the mouth	Stereotypies, sleeping and behavioral disturbances	Anemia, dextrocardia,	EEG until 3.5 months: focal spikes on a normal background; normal aspect at 1-year-old	Brain MRI: inferior vermis hypoplasia, cisterna magna, foliar distortion of the cerebellum, hypothalamic fusion anomaly	Phenobarbital, valproate	Longest seizure-free interval: 6 months
Olson et al. [7], 2018	Case series (Case 3)	Male/4 days	Not described	15 years	Delayed motor acquisition	Synophris, hypertelorism, down-slanting palpebral fissures, thin upper lip, down-turned corners of the mouth, prominent incisors and widely-spaced teeth, everted vermillion of lower lip	Speech delay	Syndactyly of toes, cryptorchidism	EEG: not available	Brain MRI: increased subarachnoid spaces	Carbamazepine	Not described
Olson et al. [7], 2018	Case series (Case 4)	Female/7 days	Not described	8 years	Strabismus, delayed motor acquisition	Synophris, hypertelorism, down-slanting palpebral fissures, broad nasal root, thin upper lip, down-turned corners of the mouth, prominent incisors	Speech delay, mild autistic disorder	Anemia	EEG in neonatal period: multifocal sharp waves, intermittent generalized burst of epileptic activity	Brain MRI: inferior vermis hypoplasia, mega cisterna magna, mild distortion of the cerebellar folia	Phenobarbital, pyridoxal-5-phosphate, pyridoxine, valproate	2 years seizure-free interval on antiepileptic treatment, reoccurrence of seizures at tempted withdrawal of valproate
Olson et al. [7], 2018	Case series (Case 5)	Male/2 days	Clonic and generalized tonic-clonic seizures	19 months	Strabismus, visual problems, delayed motor skills,	Broad nasal root, thin upper lip, down-turned corners of the lips	Not described	Anemia, V finger clinodactyly, variant transverse palmar crease	EEG in the neonatal period: normal; 4 months: generalized slowing and multifocal sharp waves; frequent focal seizures	Brain MRI: inferior vermis hypoplasia, mega cisterna magna, foliar distortion of the left cerebellar hemisphere, hypothalamic fusion anomaly	Levetiracetam, phenobarbital, carbamazepine	The longest seizure-free interval: 9 months
Olson et al. [7], 2018	Case series (Case 6)	Male/2 days	Not described	8 years	Strabismus, astigmatism, myopia, anisocoria, delayed motor acquisitions,	Thin upper lip, down-slanting palpebral fissures, everted vermillion of lower lip	Obsessive compulsive disorder	Finger pads, small ventricular septal defects, testis ectopia	EEG in the neonatal period: left temporal spikes;	Brain MRI performed at 10 days and 4 months: no pathological findings	Topiramate	The longest seizure-free interval: 3.5 year
Olson et al. [7], 2018	Case series (Case 7)	Male/2 days	Focal tonic stiffening, later clonic seizures, autonomic features	16 months	Mild conductive hearing loss, poor feeding, axial hypotonia, unable to walk, slightly increased tone in hands	Broad nasal root, thin upper lip	Speech delay	Mild anemia, café au lait birthmark, frequent ear and respiratory infections, cryptorchidism	EEG at 6–7 weeks: epileptiform activity; 9 months: normal aspect	Brain MRI: inferior vermis hypoplasia, left retro cerebellar cysts, distortion of the left cerebellar hemisphere	Phenobarbital	Not described
Olson et al. [7], 2018	Case series (Case 8)	Female/2 weeks	Focal, latter tonic seizures	5 years	Hypermetropic astigmatism, unable to walk	Hypertelorism, broad nasal root, thin upper lip, down-slanting palpebral fissures	Atypical social and behavioral features	Not described	EEG at 6 weeks: right frontocentral and left temporal subtle aberration	Brain MRI in the neonatal period: normal aspect	Phenobarbital, sodium valproate	Seizure-free for 3.5 year
Olson et al. [7], 2018	Case series (Case 9)	Female/2 days of life	Focal tonic, tonic-clonic, myoclonic; in evolution generalized tonic-clonic and tonic seizures	3 years	Myopia, astigmatism, cortical visual impairment, delayed motor skills, diffuse hypotonia	Synophris, broad nasal root, thin upper lip	Atypical social and behavioral features	Accessory caudally placed nipples	EEG in the neonatal period: excess discontinuity and multifocal sharp waves; EEG at the age of 2: intermittent generalized slowing, intermittent left temporal slowing	Brain MRI: mega cisterna magna, cerebellar foliar distortion, hypothalamic fusion anomaly	Levetiracetam, phenobarbital	The longest seizure-free interval under treatment: 2 years
Olson et al. [7], 2018	Case series (Case 10)	Male/1–2 months	Eye deviation and clonic seizure, later in evolution generalized tonic-clonic seizure	7 years	Delayed motor acquisitions, diffuse hypotonia	Mildly dysmorphic	Autism spectrum disorder	Bilateral palmar crease	EEG at 4 months: normal; EEG at 17 months: rare generalized spikes	Brain MRI: mega cisterna magna	Levetiracetam	The longest seizure-free interval: 2 years
Olson et al. [7], 2018	Case series (Case 11)	Male/1 day	Focal seizures until 2 months of age; generalized tonic-clonic seizures latter in evolution	12.5 years	Hypermetropic astigmatism, transient nystagmus, delay motor skills, speech delay	Broad nasal root, thin upper lip, down-turned corners of the lips, widely-spaced teeth	Autism spectrum disorder	V finger brachyclino-dactyly, broad and tapering short fingers; frequent otitis; micro penis, unilateral cryptorchidism, velo pharyngeal hypotonia, central precocious puberty, infantile hypertrophic pyloric stenosis	EEG in the neonatal period: epileptic discharges in the left Rolandic region	Brain MRI: thick corpus callosum, inferior vermis hypoplasia	Valproate	Not described
Olson et al. [7], 2018	Case series (Case 12)	Female/3 days	Tonic and focal tonic-clonic; status epilepticus	9 months	Hypotonia	Hypertelorism, thin upper lip, down-turned corners of the lips, prominent incisors	Transient stereotypes	V finger clinodactyly, brachycephaly, inverted nipples	EEG in the neonatal period: sharp waves and excess multifocal spikes more evident in the bilateral temporal regions; 2 months: high amplitude background, multifocal spikes	Brain MRI: mega cisterna magna, severe foliar distortion of the cerebellum	Levetiracetam phenobarbital oxcarbazepine	Not described
Olson et al. [7], 2018	Case series (Case 13)	Female/2 weeks	Focal tonic-clonic seizures; latter in evolution: focal or generalized seizures	3.5 years	Delayed motor acquisition, axial hypotonia, increased tendon reflexes	Hypertelorism, thin upper lip, down-turned corners of the lips, down-turned corners of the lips, everted vermillion of lower lip	Stereotypes, severe speech delay	Atrial septum defect, sacral pit	EEG: normal at six days; EEG in the neonatal period: multifocal epileptic activity, slow spikes with high amplitude; EEG at 17 months: spikes at vertex, enhanced in sleep	Brain MRI: cerebellar foliar distortion	Vigabatrin, levetiracetam, pyridoxal phosphate, pyridoxine, lamotrigine, valproate, clobazam	Not described
Olson et al. [7], 2018	Case series (Case 14)	Female/3 days	Tonic and later in evolution also generalized tonic-clonic seizures	8 years	Delayed motor acquisition, diffuse hypotonia, wide-based gait,	Broad nasal root, thin upper lip, everted vermillion of lower lips	Speech delay, selective mutism	V finger clinodactyly, right transverse palmar crease	EEG in the neonatal period: bilateral central and temporal sharp waves; EEG at 10 months: left frontocentral spikes; EEG at 22 months: spike or poly spike waves with high amplitude; EEG at 3 years: generalized slowing, left temporal epileptic discharges	Brain MRI: mild subarachnoid hemorrhage; prominent cisterna magna; slight foliar distortion of the left cerebellar hemisphere	Phenobarbital, pyridoxine, levetiracetam lacosamide	Not described
Dentici et al. [1], 2019	Case report	Male/3 days of life	Impairment of consciousness, upward rolling of the eyes	7 years	Delayed global motor skills during childhood; dysphagia, difficult chewing	Relative macrocephaly, broad and highly arched eyebrows, long eyelashes, broad nasal tip, rotated years, thin upper lip, spaced teeth, down-turned corners of the lips	Limited verbal speech; reduced intelligence quotient (IQ) (47)	Reduced growth velocity (required GH treatment)	Epileptic abnormalities in central region, bilaterally; Normal EEG at 7 years	Brain MRI: abnormal cerebellar foliation pattern in the posterior and basal portions of the right hemisphere; white matter reduction in the parietal regions and in the periventricular posterior regions, lateral ventricles asymmetry, centrum semiovale hyperintensities in the frontal regions	Phenobarbital; Vigabatrin and valproic acid	with seizures remission for one month; recurrence with blank staring, limb hypertonia, incontrollable crying; good control of seizures; antiepileptic drug discontinuation at 6 years
Terrone et al. [15], 2020	Case report	Female/48 h of life	Brief, tonic seizures of the upper limbs accompanied by autonomic features (facial flushing, perioral cyanosis); at 4 months: oro masticatory automatism, eye staring, dystonic postures of limb and trunk	4 months	Truncal hypotonia	Hypertelorism, broad nasal root, down-turned corners of the mouth, down-slanting palpebral fissures	Developmental delay in language and social communication	Monoarticular arthritis of the left knee	Interictal EEG: Multifocal epileptic abnormalities on the anterior and central areas of the right hemisphere; ictal EEG: right hemisphere focal seizures with rapid generalization	Brain MRI: enlargement of the left posterior horn of the lateral ventricle, irregular hemispheric cerebellar foliation	Phenobarbital, levetiracetam; Carbamazepine was added with gradual withdrawn of phenobarbital	3 months seizures free; Recurrence of seizures at 4 months; At 20 months of life: seizures free with carbamazepine
Sakaguchi Y et al. [16], 2020	Case report	Male/2 months	Tonic seizures	23-year-old	Hypotonia during neonatal period, delayed head control, delayed motor skills; upper visual field defect in his left eye; left ear sensorineural deafness; adult life: diffuse hypotonia, wide-based gait	Irideal and choroidal coloboma in the inferior quadrant of the left eye, astigmatism; highly arched eyebrows, down-slanting palpebral fissures, bilateral mild ptosis, broad nasal root, low-set ears	Delayed speaking and language comprehension	Pectus excavates, slender fingers, talipes plantaris	EEG: epileptic discharges in the right occipital region;	Brain MRI: dysgenesis and progressive atrophy of the cerebellum, foliar distortion of the inferior hemisphere more evident on the left side; hyperplasia of the anterior pituitary gland	Diazepam (ineffective), phenobarbital	Controlled seizures at 6 months; medication free since the age of 9; able to attend college
Valenzuela I et al. [17], 2021	Case report	Male/3 days after birth	Hypertonic seizures with generalized cyanosis	25-year-old	Hypotonia and hypoactivity evident since birth; mild global developmental delay with delayed motor skills; oropharyngeal dysphagia diagnosed at 20 years	Widely-spaced eyes, down slanted palpebral fissure, strabismus, laterally extended thick eyebrows, retrognathia, thick upper lip, high palate, low-set ears	Mild global developmental delay, reduced cognitive performance	Left cryptorchidism, pectus carinatum, distal joint laxity, slender fingers; progressive congestive heart failure with idiopathic dilated cardiomyopathy diagnosed at 14-years old	Not available/not described by the authors	Brain MRI: severe dysmorphic colpocephalic lateral ventriculomegaly with chronic hydrocephalus considered to be secondary to the vascular malformation; vein of Galen aneurysm present	Levetiracetam	Seizure-free under treatment with levetiracetam; stable under treatment for cardiac failure
Mizuno T et al. [18], 2021	Case series (Case 1)	Female/2 weeks after birth	Frequent tonic convulsions with head turning	2-year-old	Hypotonia, mild psychomotor developmental delay	Hypertelorism, downward-slanting palpebral fissures, thin upper lip, down-turned corners of the mouth, broad nasal root	Mild psychomotor developmental delay	Not described	High-voltage slow waves in the left posterior-temporal areas	Brain MRI: decreased posterior periventricular white matter volume, old subependymal hemorrhage (perinatal injury)	Carbamazepine, clobazam	Almost controlled at 2 years of age
Mizuno T et al. [18], 2021	Case series (Case 2)	Female/3 days after birth	Focal or generalized tonic convulsions	12-years old	Hypotonia	Hypertelorism, downward-slanting palpebral fissures, thin upper lip, down-turned corners of the mouth, broad nasal root	Autism spectrum disorder, severe intellectual disability	Not described	Spikes over the right frontal areas	Brain MRI: normal findings	Topiramate, zonisamide, clonazepam; latter: lamotrigine, valproic acid, clonazepam	Controlled between 4-year-old and 9 years old; controlled latter again after medication changes
Mizuno T et al. [18], 2021	Case series (Case 3)	Female/3 days after birth	Tonic convulsions	3-year-old	Normal psychomotor development	Hypertelorism, downward-slanting palpebral fissures, everted vermillion of lower lip, broad nasal root	Normal psychomotor development	Not described	Ictal EEG (6 months): epileptic discharges in the left hemisphere	Brain MRI: right venous sinus thrombosis (at 3 days of life)	Carbamazepine	Controlled at 9 months; latter seizure-free without medication
Cesaroni E et al. [19], 2022	Case report (Case 1)	Male/7 days after birth	Focal seizures with loss of contact, staring, crying, apnea with severe cyanosis, followed by tonic and vibratory prolonged phase	11-month-old	Hypotonia, delayed motor skills	Long eyelashes, highly arched and sparse broad eyebrows, broad nasal tip, smooth philtrum, down-turned corners of the mouth, thin and everted upper lip vermillion	Delayed personal/social skills	Not described	Generalized slowing and with sporadic multifocal sharp waves Ictal EEG: focal rhythmic sharp waves, polyspike activity	Brain MRI: prominence of cisterna magna, mild inferior vermis and cerebellar hypoplasia	Phenobarbital; adjustments of the dose at 3 months; substitution with VPA and nitrazepam at 9 months; 12 moths: substitution with carbamazepine	Seizure remission with reoccurrence after 3 months, 9 months, 12 months; seizure-free at 19 months
Cesaroni E et al. [19], 2022	Case report (Case 2)	Female/a few weeks after birth	Three brief febrile tonic seizures	37-year-old	Not described	Synophyris, highly arched and sparse broad eyebrows, long eyelashes, broad nasal tip, thin everted upper lip, down-turned corners of the mouth	Learning disabilities during childhood	Not described	EEG (during childhood): reported to be normal	Brain computed tomography (CT) scan (at 3 months): no alterations	Phenobarbital	Seizure-free from 3 months of age; without medication since childhood
Sánchez-Soler et al. [20], 2021	Case report (Case 3)	Female/3 months of age	Right-sided deviation of the eyes and head, dystonic posturing of the limbs, generalized hypertonia	3-year-old	Psychomotor retardation; developmental delay; expressive language was affected	Facial dysmorphism	Occasional episodes of self and hetero-aggression, psychomotor retardation	Polydactyly in both feet	EEG at admission: diffuse background slowing, without focal abnormalities; interictal EEG at 24 and 30 moths: normal results	Brain CT scan: no alterations	Midazolam and phenytoin in status epilepticus; Valproic acid; zonisamide	No response at the initial treatment with valproic acid; exacerbated the symptoms; Presented other 3 seizures (5 months of life, respectively 24 and 30 months); seizure-free until the age of 3 when the case was reported
Perulli M et al. [21], 2023	Case report	Male/8 days of life	2 months: repetitive focal to bilateral motor seizures, severe desaturation; frequent episodes of status epilepticus	12-year-old	Developmental delay	Facial dysmorphism present	Autism spectrum disorder, severe speech delay	Hand dysmorphism present	EEG (8 days): sporadic sharp waves in the anterior regions EEG (4.5 years): multifocal EEG pattern, overactivation in sleep EEG (11 years): theta background activity in awake state, no epileptiform activity	Brain MRI in the first month of life: hyperintensity in the periventricular white matter Brain MRI at 2.5 months: other changes occurred with global thickening of the corpus callosum, mega cisterna permagna	Diazepam, phenobarbital, phenytoin, levetiracetam, midazolam to whom the patient was refractory; improvement with pyridoxine, folinic acid, pyridoxal phosphate	Seizure-free since the age of 6 years, depends on his parents
Chou IJ et al. [22], 2023	Case series and literature review (case 1)	Male/2 weeks of age	Generalized tonic-clonic seizures; repetitive spasms in the left face, left arm and hand and postural tonic spasms;	NA	Gross motor delay, speech delay, hypotonia	Synophrys, wide-spaced teeth	Speech delay	Right hydronephrosis, horseshoe kidney	Focal cortical dysfunction over the right frontal area; Video EEG performed at 6 months: interictal epileptic discharges located at Cz, C4, C3 and F3	Brain ultrasonography and MRI: mega cisterna magna with prominent extracerebral cerebrospinal fluid spaces	Oral diazepam for the first seizures for 1 month; Phenobarbital and oxcarbazepine; Intravenous pyridoxal phosphate then oral inactive vitamin B6; ketogenic diet latter discontinued for dietary intolerance; levetiracetam was added	Seizure-free until 3 months of age; Seizures recurrence at 3 months in febrile context (pneumonia); Recurrence of seizure at the age of 6 months: effective control of seizures
Chou IJ et al. [22], 2023	Case series and literature review (case 2)	Female/1 month of age	Grasping, tonic trunk posture; tonic movements in all upper and lower limbs, cyanosis of the lips, facial flushing, upward gaze, postictal lethargy; latter in evolution tonic-clonic seizures	NA	Gross motor and speech delay;	Facial dysmorphism, hypertelorism	Speech delay; attention deficit hyperactivity disorder	Repetitive urinary infections; right hydronephrosis, bilateral vesicoureteral reflux, atrial septal defect	Initial interictal EEG: slow waves in both hemispheres, focal spikes in the bilateral centrotemporal areas; After 3 years: generalized spikes; in evolution: interictal EEG: activity in the right centrotemporal area; normal EEG at 10 years of age under treatment	Brain MRI: vermis hypoplasia, mega cisterna magna; MRI at 7 years: mild prominence of the sulci and cisterns in both cerebral hemispheres, left mesial temporal sclerosis	Intravenous pyridoxal phosphate, shifted latter to oral vitamin B6; Oxcarbazepine was associated; Levetiracetam and valproic acid were added	3 years seizure-free; Recurrence of tonic-clonic seizures
Chou IJ et al. [22], 2023	Case series and literature review (case 3)	Female/2 weeks of age	Screaming, facial flush, general muscle tightening; focal seizure with: upward gaze, eye deviation to the left, dilated pupils without light reflex, head turning to the right, jerking of the left limbs, oxygen desaturation (increased frequency of the focal seizures that occurred between generalized seizures: up to 20/day); other seizures: yawning, hiccupping, bicycling movements of the legs, grasping, facial flushing	NA	Poor sucking and feeding; Global developmental delay; hypotonia, horizontal nystagmus, microcephaly; failure to thrive	Facial dysmorphism	Global developmental delay	Gastroesophageal reflux disease; atrial septal defect	Initial 24-h video EEG: diffuse cortical dysfunction, multifocal epileptiform discharges; Follow-up EEG: similar findings with the initial EEG	Brain MRI: mega cisterna magna	Phenobarbital; Levetiracetam and intravenous pyridoxal phosphate were added (shifted later on oral vitamin B6); Later in evolution a combination of vigabatrin, phenobarbital, clonazepam	Seizure persistence The seizure frequency was reduced to once or twice monthly
Checri R et al. [23], 2023	Case report	Male/10 months	Asymmetrical epileptic spasms	7-year-old child	Developmental delay with hypotonia, poor sucking, left hemiparesis	Round face with hypertelorism, oblique downward and outward palpebral clefts, wide nose, small teeth widely-spaced	Mild cognitive disabilities	Short extremities	Multifocal spikes associated with 1–2 Hz slow waves	Brain MRI: right insular polymicrogyria and pachygyria, cerebellar dysgenesis with disorganization of the cerebellar foliation mostly in the vermis	Resistant to prednisolone, vigabatrin, valproate, levetiracetam and lamotrigine; cessation of epileptic seizures after radiprodil (allosteric modulator of NMDA receptors) association	Seizure-free at 7 years on monotherapy (valproate)
Stoian et al. (our own case)	Case report	Male/4.5 months	Tonic seizures; later in evolution a left facial hemi spasm	31 years	Reduced visual acuity (myopia), one and a half syndrome with left abduction nystagmus, left facial hemi spasms, divergent strabismus, flaccid tetra paresis, bilateral inconstant Babinski sign, gait instability, truncal ataxia,	Mild facial dysmorphism with thin upper lip and slightly spaced teeth	Social isolation, bradypsychia, bradylalia, echolalia, minimal linguistic baggage, behavioral stereotypes, MMSE17 points	Cryptorchidism	EEG: Poor unmodulated alpha activity, frontal intermittent theta activity; sharp waves and sharp peaks on the left central, parietal and temporal derivations	Brain CT: generalized sufferance pf white matter; cavum septum pellucidi, enlarged ventricular system, cerebral and cerebellar atrophy. Brain MRI performed before hospital admission: severe cerebral and cerebellar atrophy, vermis hypoplasia, supratentorial and infratentorial demyelinating lesions, cavum septum pellucidi, severe enlargement of the CSF spaces	Valproic acid, later replaced with levetiracetam and after a while with brivaracetam	The seizures disappeared under treatment being seizure-free since childhood; a left facial hemi spasm occurred lately

## Abbreviations

Abbreviation	Meaning
CK2A1	casein kinase 2 alpha 1
CNS	central nervous system
COVID-19	corona virus disease 2019
CSF	cerebrospinal fluid
CT	computed tomography
CTR	C-terminal region
DEE	developmental and epileptic encephalopathies
DNA	deoxyribonucleic acid
EEG	electroencephalogram
EMG	electromyography
ER	endoplasmic reticulum
FLAIR	fluid attenuated inversion recovery
FBR	furin binding region
IQ	intelligence quotient
LP	lumbar puncture
MAMs	mitochondria-associated membranes
MMSE	mini-mental state examination
MR	middle region
MRC	Medical Research Council scale
MRI	magnetic resonance imaging
NIH	National Institute of Health
PACS2	phosphofurin acidic cluster sorting protein 2
PRISMA	Preferred Reporting Items for Systematic Review and Meta-Analyses
TNF	tumor necrosis factor
TRAIL	TNF-related apoptosis-inducing ligand

## References

[1] Dentici M.L., Barresi S., Niceta M., Ciolfi A., Trivisano M., Bartuli A., Digilio M.C., Specchio N., Dallapiccola B., Tartaglia M. (2019). Expanding the clinical spectrum associated with *PACS2* mutations. Clin. Genet..

[2] Eltze C.M., Chong W.K., Cox T., Whitney A., Cortina-Borja M., Chin R.F., Scott R.C., Cross J.H. (2013). A population-based study of newly diagnosed epilepsy in infants. Epilepsia.

[3] von Deimling M., Helbig I., Marsh E.D. (2017). Epileptic Encephalopathies-Clinical Syndromes and Pathophysiological Concepts. Curr. Neurol. Neurosci. Rep..

[4] Raga S., Specchio N., Rheims S., Wilmshurst J.M. (2021). Developmental and epileptic encephalopathies: Recognition and approaches to care. Epileptic Disord..

[5] Scheffer I.E., Berkovic S., Capovilla G., Connolly M.B., French J., Guilhoto L., Hirsch E., Jain S., Mathern G.W., Moshé S.L. (2017). ILAE classification of the epilepsies: Position paper of the ILAE Commission for Classification and Terminology. Epilepsia.

[6] Scheffer I.E., French J., Hirsch E., Jain S., Mathern G.W., Moshé S.L., Perucca E., Tomson T., Wiebe S., Zhang Y.H. (2016). Classification of the epilepsies: New concepts for discussion and debate-Special report of the ILAE Classification Task Force of the Commission for Classification and Terminology. Epilepsia Open.

[7] Olson H.E., Jean-Marçais N., Yang E., Heron D., Tatton-Brown K., van der Zwaag P.A., Bijlsma E.K., Krock B.L., Backer E., Kamsteeg E.J. (2018). A Recurrent De Novo *PACS2* Heterozygous Missense Variant Causes Neonatal-Onset Developmental Epileptic Encephalopathy, Facial Dysmorphism, and Cerebellar Dysgenesis. Am. J. Hum. Genet..

[8] Han X., Deng J., Chen C., Wang X., Fang F., Li H., Luo J., Wu J. (2022). Developmental and Epileptic Encephalopathy 76: Case Report and Review of Literature. Children.

[9] Simmen T., Aslan J.E., Blagoveshchenskaya A.D., Thomas L., Wan L., Xiang Y., Feliciangeli S.F., Hung C.H., Crump C.M., Thomas G. (2005). PACS-2 controls endoplasmic reticulum-mitochondria communication and Bid-mediated apoptosis. EMBO J..

[10] Köttgen M., Benzing T., Simmen T., Tauber R., Buchholz B., Feliciangeli S., Huber T.B., Schermer B., Kramer-Zucker A., Höpker K. (2005). Trafficking of TRPP2 by PACS proteins represents a novel mechanism of ion channel regulation. EMBO J..

[11] Adzhubei I.A., Schmidt S., Peshkin L., Ramensky V.E., Gerasimova A., Bork P., Kondrashov A.S., Sunyaev S.R. (2010). A method and server for predicting damaging missense mutations. Nat. Methods.

[12] Thomas G., Aslan J.E., Thomas L., Shinde P., Shinde U., Simmen T. (2017). Caught in the act—Protein adaptation and the expanding roles of the PACS proteins in tissue homeostasis and disease. J. Cell Sci..

[13] Zhang H., Gao K., Wang S., Zhang Y.H., Yang Z.X., Wu Y., Jiang Y.W. (2023). PACS gene family-related neurological diseases: Limited genotypes and diverse phenotypes. World J. Pediatr..

[14] Aslan J.E., You H., Williamson D.M., Endig J., Youker R.T., Thomas L., Shu H., Du Y., Milewski R.L., Brush M.H. (2009). Akt and 14-3-3 control a PACS-2 homeostatic switch that integrates membrane traffic with TRAIL-induced apoptosis. Mol. Cell.

[15] Terrone G., Marchese F., Vari M.S., Severino M., Madia F., Amadori E., Del Giudice E., Romano A., Gennaro E., Zara F. (2020). A further contribution to the delineation of epileptic phenotype in PACS2-related syndrome. Seizure.

[16] Sakaguchi Y., Yoshihashi H., Uehara T., Miyama S., Kosaki K., Takenouchi T. (2021). Coloboma may be a shared feature in a spectrum of disorders caused by mutations in the WDR37-PACS1-PACS2 axis. Am. J. Med. Genet. A.

[17] Valenzuela I., Guillén Benítez E., Sanchez-Montanez A., Limeres J., López-Grondona F., Cuscó I., Tizzano E.F. (2022). Vein of Galen aneurysm, dilated cardiomyopathy, and slender habitus in a patient with a recurrent pathogenic variant in *PACS2*. Am. J. Med. Genet. A.

[18] Mizuno T., Miyata R., Hojo A., Tamura Y., Nakashima M., Mizuguchi T., Matsumoto N., Kato M. (2021). Clinical variations of epileptic syndrome associated with *PACS2* variant. Brain Dev..

[19] Cesaroni E., Matricardi S., Cappanera S., Marini C. (2022). First reported case of an inherited *PACS2* pathogenic variant with variable expression. Epileptic Disord..

[20] Sánchez-Soler M.J., Serrano-Antón A.T., López-González V., Guillén-Navarro E. (2021). New case with the recurrent c.635G>A pathogenic variant in the *PACS2* gene: Expanding the phenotype. Neurologia.

[21] Perulli M., Picilli M., Contaldo I., Amenta S., Gambardella M.L., Quintiliani M., Musto E., Turrini I., Veredice C., Zollino M. (2023). Pyridoxine supplementation in *PACS2*-related encephalopathy: A case report of possible precision therapy. Seizure.

[22] Chou I.J., Hou J.Y., Fan W.L., Tsai M.H., Lin K.L. (2023). Long-Term Outcome of Neonatal Seizure with *PACS2* Mutation: Case Series and Literature Review. Children.

[23] Checri R., Dozières-Puyravel B., Elmaleh-Bergès M., Verloes A., Auvin S. (2023). *PACS2* pathogenic variant associated with malformation of cortical development and epilepsy. Epileptic Disord..

[24] Moulis M., Grousset E., Faccini J., Richetin K., Thomas G., Vindis C. (2019). The Multifunctional Sorting Protein PACS-2 Controls Mitophagosome Formation in Human Vascular Smooth Muscle Cells through Mitochondria-ER Contact Sites. Cells.

[25] https://www.ncbi.nlm.nih.gov/clinvar.

[26] Kopanos C., Tsiolkas V., Kouris A., Chapple C.E., Albarca Aguilera M., Meyer R., Massouras A. (2019). VarSome: The human genomic variant search engine. Bioinformatics.

[27] Pejaver V., Urresti J., Lugo-Martinez J., Pagel K.A., Lin G.N., Nam H.J., Mort M., Cooper D.N., Sebat J., Iakoucheva L.M. (2020). Inferring the molecular and phenotypic impact of amino acid variants with MutPred2. Nat. Commun..

[28] http://www.ensembl.org/Homo_sapiens/Tools/VEP.

[29] https://www.ncbi.nlm.nih.gov/medgen/1648486.

[30] Stoian A., Bajko Z., Maier S., Cioflinc R.A., Grigorescu B.L., Moțățăianu A., Bărcuțean L., Balașa R., Stoian M. (2021). High-dose intravenous immunoglobulins as a therapeutic option in critical illness polyneuropathy accompanying SARS-CoV-2 infection: A case-based review of the literature (Review). Exp. Ther. Med..

[31] Amore G., Spoto G., Ieni A., Vetri L., Quatrosi G., Di Rosa G., Nicotera A.G. (2021). A Focus on the Cerebellum: From Embryogenesis to an Age-Related Clinical Perspective. Front. Syst. Neurosci..

[32] Divya K.P., Kishore A. (2020). Treatable cerebellar ataxias. Clin. Park. Relat. Disord..

[33] Maier S., Moțățăianu A., Bărcuțean L., Balint A., Huțanu A., Bajko Z., Stoian A., Andone S., Bălașa R. (2020). Interferon β 1A, an immunomodulator in relapsing remitting multiple sclerosis patients: The effect on pro inflammatory cytokines. Farmacia.

[34] Stoian A., Bajko Z., Stoian M., Cioflinc R.A., Niculescu R., Arbănași E.M., Russu E., Botoncea M., Bălașa R. (2023). The Occurrence of Acute Disseminated Encephalomyelitis in SARS-CoV-2 Infection/Vaccination: Our Experience and a Systematic Review of the Literature. Vaccines.

[35] Stoian A., Stoian M., Bajko Z., Maier S., Andone S., Cioflinc R.A., Moțățăianu A., Barcuțean L., Bălașa R. (2022). Autoimmune Encephalitis in COVID-19 Infection: Our Experience and Systematic Review of the Literature. Biomedicines.

[36] Balasa R., Maier S., Barcutean L., Stoian A., Motataianu A. (2020). The direct deleterious effect of Th17 cells in the nervous system compartment in multiple sclerosis and experimental autoimmune encephalomyelitis: One possible link between neuroinflammation and neurodegeneration. Rev. Română Med. Lab..

[37] Gorodetsky C., Fasano A. (2022). Developmental and Epileptic Encephalopathies in Adults: An Evolving Field. Neurology.

[38] Winkler E.A., Nishida Y., Sagare A.P., Rege S.V., Bell R.D., Perlmutter D., Sengillo J.D., Hillman S., Kong P., Nelson A.R. (2015). GLUT1 reductions exacerbate Alzheimer’s disease vasculo-neuronal dysfunction and degeneration. Nat. Neurosci..

[39] Youker R.T., Shinde U., Day R., Thomas G. (2009). At the crossroads of homoeostasis and disease: Roles of the PACS proteins in membrane traffic and apoptosis. Biochem. J..

[40] Schuurs-Hoeijmakers J.H., Oh E.C., Vissers L.E., Swinkels M.E., Gilissen C., Willemsen M.A., Holvoet M., Steehouwer M., Veltman J.A., De Vries B.B. (2012). Recurrent de novo mutations in PACS1 cause defective cranial-neural-crest migration and define a recognizable intellectual-disability syndrome. Am. J. Hum. Genet..

[41] Neagu A.C., Budișteanu M., Gheorghe D.C., Mocanu A.I., Mocanu H. (2022). Rare Gene Mutations in Romanian Hypoacusis Patients: Case Series and a Review of the Literature. Medicina.

[42] Wan L., Molloy S.S., Thomas L., Liu G., Xiang Y., Rybak S.L., Thomas G. (1998). PACS-1 defines a novel gene family of cytosolic sorting proteins required for trans-Golgi network localization. Cell.

[43] Li C., Li L., Yang M., Zeng L., Sun L. (2020). PACS-2: A key regulator of mitochondria-associated membranes (MAMs). Pharmacol. Res..

[44] Pinton P. (2018). Mitochondria-associated membranes (MAMs) and pathologies. Cell Death Dis..

[45] Area-Gomez E., de Groof A., Bonilla E., Montesinos J., Tanji K., Boldogh I., Pon L., Schon E.A. (2018). A key role for MAM in mediating mitochondrial dysfunction in Alzheimer disease. Cell Death Dis..

[46] Zang R.X., Mumby M.J., Dikeakos J.D. (2022). The Phosphofurin Acidic Cluster Sorting Protein 2 (PACS-2) E209K Mutation Responsible for PACS-2 Syndrome Increases Susceptibility to Apoptosis. ACS Omega.

[47] Wieckowski M.R., Giorgi C., Lebiedzinska M., Duszynski J., Pinton P. (2009). Isolation of mitochondria-associated membranes and mitochondria from animal tissues and cells. Nat. Protoc..

[48] Perrone M., Caroccia N., Genovese I., Missiroli S., Modesti L., Pedriali G., Vezzani B., Vitto V.A.M., Antenori M., Lebiedzinska-Arciszewska M. (2020). The role of mitochondria-associated membranes in cellular homeostasis and diseases. Int. Rev. Cell Mol. Biol..

[49] Thi My Nhung T., Phuoc Long N., Diem Nghi T., Suh Y., Hoang Anh N., Jung C.W., Minh Triet H., Jung M., Woo Y., Yoo J. (2023). Genome-wide kinase-MAM interactome screening reveals the role of CK2A1 in MAM Ca^2+^ dynamics linked to DEE66. Proc. Natl. Acad. Sci. USA.

[50] Giacomello M., Pellegrini L. (2016). The coming of age of the mitochondria-ER contact: A matter of thickness. Cell Death Differ..

[51] Kravic B., Harbauer A.B., Romanello V., Simeone L., Vögtle F.N., Kaiser T., Straubinger M., Huraskin D., Böttcher M., Cerqua C. (2018). In mammalian skeletal muscle, phosphorylation of TOMM22 by protein kinase CSNK2/CK2 controls mitophagy. Autophagy.

[52] Afzal M., Kren B.T., Naveed A.K., Trembley J.H., Ahmed K. (2020). Protein kinase CK2 impact on intracellular calcium homeostasis in prostate cancer. Mol. Cell. Biochem..

[53] Zeng K.W., Wang J.K., Wang L.C., Guo Q., Liu T.T., Wang F.J., Feng N., Zhang X.W., Liao L.X., Zhao M.M. (2021). Small molecule induces mitochondrial fusion for neuroprotection via targeting CK2 without affecting its conventional kinase activity. Signal Transduct. Target. Ther..

[54] Lynes E.M., Raturi A., Shenkman M., Ortiz Sandoval C., Yap M.C., Wu J., Janowicz A., Myhill N., Benson M.D., Campbell R.E. (2013). Palmitoylation is the switch that assigns calnexin to quality control or ER Ca^2+^ signaling. J. Cell Sci..

[55] Bravo-Sagua R., Parra V., López-Crisosto C., Díaz P., Quest A.F., Lavandero S. (2017). Calcium Transport and Signaling in Mitochondria. Compr. Physiol..

[56] Bagur R., Hajnóczky G. (2017). Intracellular Ca^2+^ Sensing: Its Role in Calcium Homeostasis and Signaling. Mol. Cell.

[57] Porter R.J., Dhir A., Macdonald R.L., Rogawski M.A. (2012). Mechanisms of action of antiseizure drugs. Handb. Clin. Neurol..

[58] Johnstone R.W., Frew A.J., Smyth M.J. (2008). The TRAIL apoptotic pathway in cancer onset, progression and therapy. Nat. Rev. Cancer.

[59] Aslan J.E., Thomas G. (2009). Death by committee: Organellar trafficking and communication in apoptosis. Traffic.

[60] Feliciangeli S.F., Thomas L., Scott G.K., Subbian E., Hung C.H., Molloy S.S., Jean F., Shinde U., Thomas G. (2006). Identification of a pH sensor in the furin propeptide that regulates enzyme activation. J. Biol. Chem..

[61] Betz C., Stracka D., Prescianotto-Baschong C., Frieden M., Demaurex N., Hall M.N. (2013). Feature Article: mTOR complex 2-Akt signaling at mitochondria-associated endoplasmic reticulum membranes (MAM) regulates mitochondrial physiology. Proc. Natl. Acad. Sci. USA.

[62] Werneburg N.W., Bronk S.F., Guicciardi M.E., Thomas L., Dikeakos J.D., Thomas G., Gores G.J. (2012). Tumor necrosis factor-related apoptosis-inducing ligand (TRAIL) protein-induced lysosomal translocation of proapoptotic effectors is mediated by phosphofurin acidic cluster sorting protein-2 (PACS-2). J. Biol. Chem..

[63] Guicciardi M.E., Werneburg N.W., Bronk S.F., Franke A., Yagita H., Thomas G., Gores G.J. (2014). Cellular inhibitor of apoptosis (cIAP)-mediated ubiquitination of phosphofurin acidic cluster sorting protein 2 (PACS-2) negatively regulates tumor necrosis factor-related apoptosis-inducing ligand (TRAIL) cytotoxicity. PLoS ONE.

[64] Atkins K.M., Thomas L.L., Barroso-González J., Thomas L., Auclair S., Yin J., Kang H., Chung J.H., Dikeakos J.D., Thomas G. (2014). The multifunctional sorting protein PACS-2 regulates SIRT1-mediated deacetylation of p53 to modulate p21-dependent cell-cycle arrest. Cell Rep..

[65] Urbanska M., Gozdz A., Swiech L.J., Jaworski J. (2012). Mammalian target of rapamycin complex 1 (mTORC1) and 2 (mTORC2) control the dendritic arbor morphology of hippocampal neurons. J. Biol. Chem..

[66] Toyo-oka K., Wachi T., Hunt R.F., Baraban S.C., Taya S., Ramshaw H., Kaibuchi K., Schwarz Q.P., Lopez A.F., Wynshaw-Boris A. (2014). 14-3-3ε and ζ regulate neurogenesis and differentiation of neuronal progenitor cells in the developing brain. J. Neurosci..

[67] Pisano T., Numis A.L., Heavin S.B., Weckhuysen S., Angriman M., Suls A., Podesta B., Thibert R.L., Shapiro K.A., Guerrini R. (2015). Early and effective treatment of KCNQ2 encephalopathy. Epilepsia.

[68] Dilena R., Striano P., Gennaro E., Bassi L., Olivotto S., Tadini L., Mosca F., Barbieri S., Zara F., Fumagalli M. (2017). Efficacy of sodium channel blockers in SCN2A early infantile epileptic encephalopathy. Brain Dev..

[69] Mallik S., Kundu S. (2018). Topology and Oligomerization of Mono- and Oligomeric Proteins Regulate Their Half-Lives in the Cell. Structure.

[70] Yamaguchi Y., Miura M. (2015). Programmed cell death in neurodevelopment. Dev. Cell.

[71] Blanquie O., Kilb W., Sinning A., Luhmann H.J. (2017). Homeostatic interplay between electrical activity and neuronal apoptosis in the developing neocortex. Neuroscience.

[72] Cocito C., Merighi A., Giacobini M., Lossi L. (2016). Alterations of Cell Proliferation and Apoptosis in the Hypoplastic Reeler Cerebellum. Front. Cell. Neurosci..

[73] Holder J.L., Lotze T.E., Bacino C., Cheung S.W. (2012). A child with an inherited 0.31 Mb microdeletion of chromosome 14q32.33: Further delineation of a critical region for the 14q32 deletion syndrome. Am. J. Med. Genet. A.

